# Effect of Cod Residual Protein Supplementation on Markers of Glucose Regulation in Lean Adults: A Randomized Double-Blind Study

**DOI:** 10.3390/nu12051445

**Published:** 2020-05-16

**Authors:** Iselin Vildmyren, Alfred Halstensen, Adrian McCann, Øivind Midttun, Per Magne Ueland, Åge Oterhals, Oddrun Anita Gudbrandsen

**Affiliations:** 1 Dietary Protein Research Group, Department of Clinical Medicine, University of Bergen, 5021 Bergen, Norway; nkjgu@uib.no; 2 K. Halstensen AS, P.O. Box 103, 5399 Bekkjarvik, Norway; alfred.halstensen@uib.no; 3 Department of Clinical Science, University of Bergen, 5021 Bergen, Norway; 4 Bevital AS, Jonas Lies veg 87, 5021 Bergen, Norway; adrian.mccann@bevital.no (A.M.); nkjbm@uib.no (Ø.M.); per.ueland@ikb.uib.no (P.M.U.); 5 Nofima, P.B. 1425 Oasen, 5844 Bergen, Norway; aage.oterhals@nofima.no

**Keywords:** dietary supplements, fish protein, marine protein, hydroxybutyrate, TMAO, biomarkers

## Abstract

Large quantities of protein-rich cod residuals, which are currently discarded, could be utilized for human consumption. Although fish fillet intake is related to beneficial health effects, little is known about the potential health effects of consuming cod residual protein powder. Fifty lean adults were randomized to consume capsules with 8.1 g/day of cod residual protein (Cod-RP) or placebo capsules (Control group) for eight weeks, in this randomized, double-blind study. The intervention was completed by 40 participants. Fasting glucose and insulin concentrations were unaffected by Cod-RP supplementation, whereas plasma concentrations of α-hydroxybutyrate, β-hydroxybutyrate and acetoacetate all were decreased compared with the Control group. Trimethylamine N-oxide concentration in plasma and urine were increased in the Cod-RP group compared with the Control group. To conclude, the reduction in these potential early markers of impaired glucose metabolism following Cod-RP supplementation may indicate beneficial glucoregulatory effects of cod residual proteins. Trimethylamine N-oxide appears to be an appropriate biomarker of cod residual protein intake in lean adults.

## 1. Introduction

Fish intake is associated with beneficial effects on risk factors for type 2 diabetes and cardiovascular disease [1,2,3,4,5,6,7], and these effects have mainly been attributed to the content of long-chain n-3 polyunsaturated fatty acids (n-3 PUFA) in fish [8]. Although underlying mechanisms are still unclear, several studies have shown that lean fish may also improve insulin sensitivity and glucose regulation, despite containing very low amounts of long-chain n-3 PUFA [9,10,11,12,13,14,15]. Small alterations in glucose regulation may be difficult to identify in short-term dietary intervention studies among healthy individuals. Therefore, other markers of glucose regulation that may be more sensitive to detecting small regulatory changes, other than fasting glucose and insulin concentrations, should be explored. Studies have shown that metabolites of fatty acid β-oxidation and ketogenic amino acid catabolism, such as α-hydroxybutyrate (α-HB), β-hydroxybutyrate (β-HB) and acetoacetate (AcAc), may reflect early changes in glucose regulation [16,17,18,19].

People in the western world mostly consume the fish fillet, and not the whole fish or the residuals. The residuals from fillet production consist of head, backbone, skin, entrails and trimmings, which contain up to 70% protein when dried [20]. Currently, large quantities of residuals from cod are discarded, and the rest is mostly utilized for non-food purposes, such as feed ingredients for agriculture and aquaculture. To our knowledge, only two clinical studies on cod residual protein intake have been conducted so far, and findings suggest that cod residual protein supplementation may beneficially affect glucose regulation and lipid metabolism [14,21]. Thus, further studies investigating potential health effects of cod residual protein for human consumption should be undertaken.

The assessment of dietary compliance is vital in nutrition intervention studies, and biomarkers reflecting dietary intake would be especially useful for evaluating intervention efficacy [22]. Higher concentrations of creatine, 1-methylhistidine (1-MeHis), 3-methylhistidine (3-MeHis) [23,24] and trimethylamine N-oxide (TMAO) [23,25,26,27,28,29,30,31] have been associated with cod fillet intake in both rats and humans. These observations highlight the potential use of these compounds as biomarkers of cod protein intake from residuals in supplement studies.

The current study aimed to investigate the effects of cod residual protein supplementation on circulating markers related to glucose regulation, and potential biomarkers of cod residual protein intake. We hypothesized that eight weeks of 8.1 g cod residual protein (Cod-RP) per day would decrease concentrations of circulating markers of glucose regulation, and increase concentrations of potential plasma and urine biomarkers of cod residual protein intake in lean adults. Cod-RP supplementation caused reduced fasting plasma concentrations of α-hydroxybutyrate, β-hydroxybutyrate and acetoacetate, compared with the Control group. Increased trimethylamine N-oxide concentrations in plasma and urine suggest it could function as a biomarker of cod residual protein intake in lean adults.

## 2. Materials and Methods

### 2.1. Participants and Ethics

The study population consisted of healthy, lean adults. The eligibility criteria included BMI ≥ 18.5 kg/m^2^ with body fat percentage 12–35% for women and 5–25% for men, stable body weight (<5 kg variation in 3 months), fasting blood glucose < 7.0 millimoles/L, and age 20–55 years. Exclusion criteria included disorders affecting kidney, heart, intestinal function, or insulin secretion, use of prescription medications for high cholesterol, seafood intake > 200 g/week, allergies towards seafood, gluten or milk, and use of dietary supplements. Pregnant and lactating women were not included in the study.

The participants provided written informed consent prior to study enrolment. The Regional Ethics Committee of Western Norway approved the study (approval no.: 2015/75). The study was conducted in accordance with the Declaration of Helsinki and is registered at clinicaltrials.gov (NCT03538834).

### 2.2. Study Design, Intervention and Protocol

This double-blind, randomized intervention study was conducted at the University of Bergen (Bergen, Norway). In brief, 50 participants were stratified according to gender and age, and randomized to one of two groups, consuming 27 capsules with a total of 8.1 g of cod residual protein (Cod-RP group), or 27 placebo capsules (Control group) daily for eight weeks. Participants were instructed to maintain their dietary and physical activity routines unchanged during the eight week intervention period. The participants attended two study visits: at baseline and after eight weeks. The study visits were performed after an overnight fast (>10 h) without consuming drink (except for water), food, medications or tobacco. Participants were instructed to not consume alcohol or engage in intensive physical activity for 24 h before the study visits.

The participants’ height was measured using a stadiometer (Telescopic Measuring Rod MZ10023-3, ADE, Hamburg, Germany), and body composition was measured using a bioelectrical impedance analyzer (InBody 720, Seoul, Korea). Participants provided urine samples in a fasted state. Fasting blood samples for whole blood and isolation of plasma were collected in Vacuette K2EDTA (Greiner Bio-One GmbH, Kremsmünster, Austria) and Vacutainer SST II Advanced Plus (Becton, Dickinson and Company, Franklin Lakes, NJ, USA) for isolation of serum. Postprandial blood samples were collected every 30 min for 2 h after the participants consumed a drink containing 75 g glucose (Dextrose from SunVita AS, Nyborg, Norway) and 35 g whey protein (Proteinfabrikken AS, Stokke, Norway) mixed with 200 mL of water, as a modified oral glucose tolerance test (OGTT).

### 2.3. Production and Analyses of Intervention Capsules

Northeast Atlantic cod (Gadus morhua) residuals from fillet production, consisting of head, backbone, skin, entrails and trimmings were processed on-board the factory trawler Granit (Halstensen Granit AS, Bekkjarvik, Norway). The cod residuals were ground, heat treated at 90 °C, pressed and dried on-board the trawler, and the solid phase and liquid phase was dried and mixed at Seagarden AS (Karmøy, Norway). All capsules for the study were produced at Pharmatech AS (Fredrikstad, Norway). A detailed description of the production of intervention capsules is described elsewhere [21] and the contents of the capsules are presented in Table 1.

Trimethylamine N-oxide (TMAO) content in the cod residual powder and placebo capsules was determined using the micro-diffusion assay described by Conway and Byrne [32] and anserine was measured by High Performance Liquid Chromatographyusing the Waters Pico-Tag method [33]. The contents of TMAO, anserine, crude protein, total fat and microorganisms were measured by Nofima BioLab (Bergen, Norway). Contents of the heavy metals cadmium and mercury were analyzed by Eurofins AS (Ålesund, Norway).

### 2.4. Analyses of Biological Samples

Glucose concentration in serum, HbA1c (glycated haemoglobin) concentration in whole blood and concentrations of creatinine and albumin in urine were analyzed on the Cobas c 111 system (Roche Diagnostics GmbH, Mannheim, Germany) using the GLUC2, A1C-3 (with A1CD2 hemolyzing reagent) and CREP2 kits from Roche Diagnostics for the c111 system. Serum insulin concentration was analyzed using the EIA-2935 ELISA kit (DRG Instruments GmbH, Marburg, Germany).

Plasma concentrations of α-HB, β-HB, AcAc, and plasma and urine concentration of 1-methylhistidine (1-MeHis, π-methyl-histidine), 3-methylhistidine (3-MeHis, τ-methyl-histidine), creatine, trimethylamine N-oxide (TMAO) and creatinine were measured by Bevital AS (http://www.bevital.no) using gas chromatography and liquid chromatography with tandem mass spectrometry [34,35]. α-HB, β-HB, AcAc, 1-MeHis, 3-MeHis and TMAO were analyzed by adding the analytes and isotope-labelled internal standards to existing assays [34,35].

### 2.5. Outcomes

The primary outcome was changes in circulating markers related to glucose regulation in lean adults after eight weeks of cod residual protein supplementation. The secondary outcome was changes in potential urine and plasma biomarkers of cod residual protein intake.

### 2.6. Sample Size

Power analysis was not feasible for the original study, which was designed as a pilot study and is the first to investigate effects of cod residuals on circulating markers of lipid metabolism in lean adults [21]. Therefore, no estimation of sample size for the current measurements was conducted prior to the study.

### 2.7. Statistical Analyses

Normality of data was assessed by the Shapiro–Wilk test, histograms and QQ plots. Variables that were not normally distributed underwent log-transformation before parametric statistical tests were performed. The Paired samples *T*-test was used to detect baseline to eight week changes within groups, and the Independent samples *T*-test was used to compare baseline to eight weekchanges between the two experimental groups. The level of statistical significance was set to *p* < 0.05. SPSS Statistics version 25 (IBM Corp. IBM SPSS for Windows, Armonk, NY, USA) was used for statistical analyses.

## 3. Results

### 3.1. Participant Characteristics

**Figure 1:**
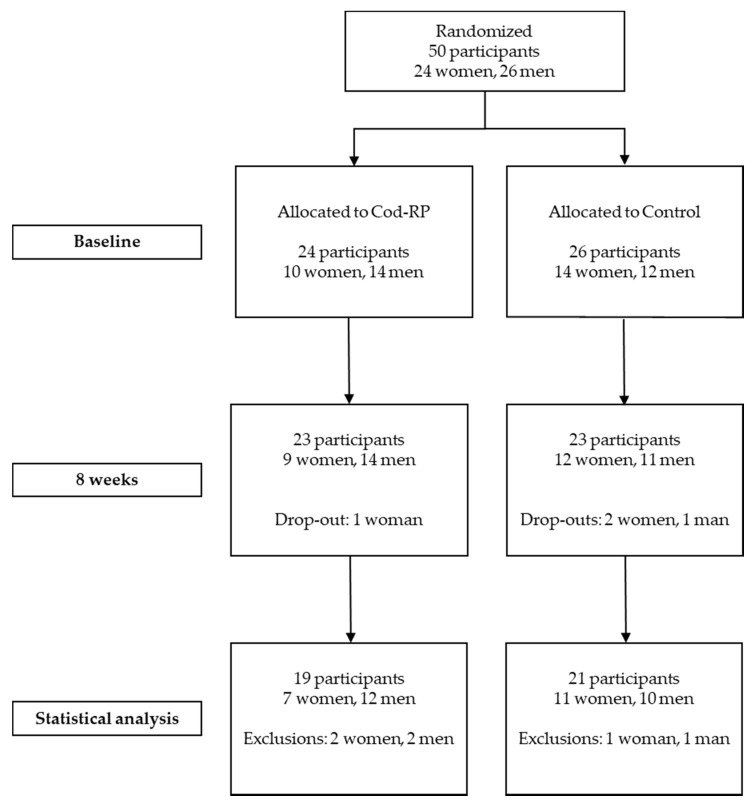
Flow of participants through the study. Participants could withdraw at any time and participants were excluded from analysis if they did not comply with the study protocol. Cod-RP, cod residual protein powder.

Of the fifty participants initially included, forty participants (18 women and 22 men) completed the eight weeks intervention (Figure 1). Four participants withdrew from the study, and six participants were excluded from further analyses due to non-compliance (*n* = 5) or disease (*n* = 1). Non-compliance was defined as not taking the study supplements, consuming > 200 g/week of seafood, and changes in habitual dietary intake or physical activity. Three participants were excluded from the OGTT due to difficulties drawing blood. Age, body weight, BMI, body fat percentage, body muscle percentage, concentrations of fasting glucose, insulin, α-HB, β-HB, AcAc and cigarette or snus use were similar between the two groups at baseline (Table 2). The concentrations of whole blood HbA1c, plasma creatinine and urine albumin (relative to creatinine) were within normal range in all participants at baseline: for HbA1c, <42 mmol/mol International Federation of Clinical Chemistry and Laboratory Medicine (IFCC); for creatinine, 45–90 µmol/L in women and 60–105 µmol/L in men; and for albumin, 0–2.5 mg/mmol. Concentrations of whole blood HbA1c, plasma creatinine and urine albumin were unchanged after eight weeks intervention (data not presented). Estimated dietary energy and macronutrient intake did not differ between the groups from baseline to eight weeks, as previously presented [21].

### 3.2. The Contents of Trimethylamine N-oxide, Anserine, Microorganisms and Heavy Metals from the Capsules

The estimated intakes of TMAO and anserine from the Cod-RP capsules were 14.4 and 7.8 mg/day, respectively (average of two measurements of the capsule composition, with deviations < 5% between parallels). TMAO and anserine were not detected in the Control capsules. The contents of microorganisms in the cod residual powder were at an acceptably low level for human consumption. It was estimated that the participants’ weekly intake of cadmium and mercury was equivalent to 0.08 and 0.13 µg/kg body weight, respectively (participants’ average body weight of 75 kg). The tolerable weekly intake for cadmium and mercury is 2.5 and 1.3 µg/kg body weight, respectively [36,37]. Thus, the estimated intake of the heavy metals cadmium and mercury from cod residual protein powder was within the tolerable weekly intake.

### 3.3. Circulating Markers Related to Glucose Regulation

**Figure 2:**
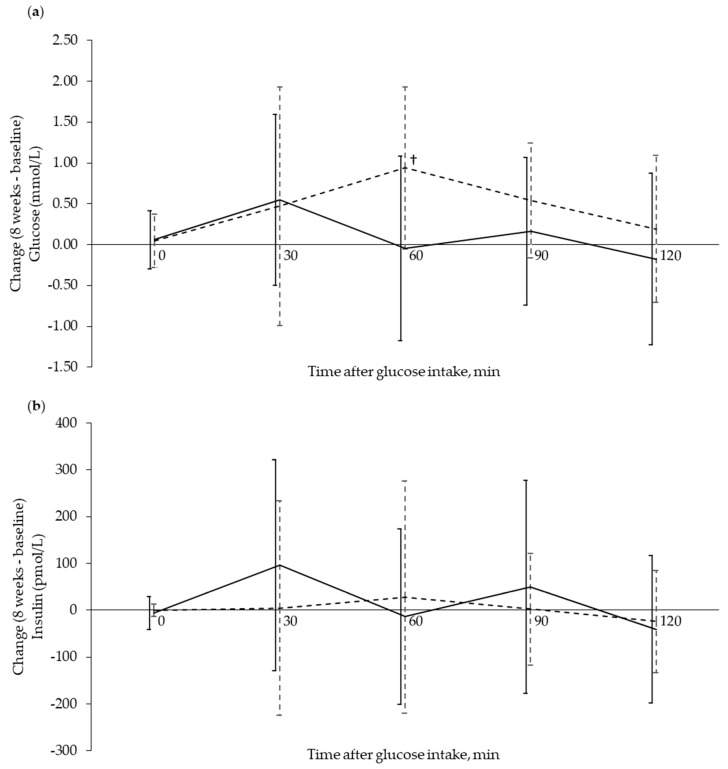
Glucose and insulin response during an oral glucose tolerance test (OGTT) shown as change from baseline to 8 weeks after supplementation. Glucose response (**a**) and insulin response (**b**) are expressed as change (8 weeks–baseline) in the Cod-RP (**―**) group and the Control group (**- -**), at 0 (fasting), 30, 60, 90 and 120 min after oral glucose intake. The results are presented as mean with standard deviation for 18 participants in the Cod-RP group and 19 participants in the Control group. † Between-group changes were compared using the independent samples *T*-test, where *p* < 0.05 was considered significant.

**Figure 3:**
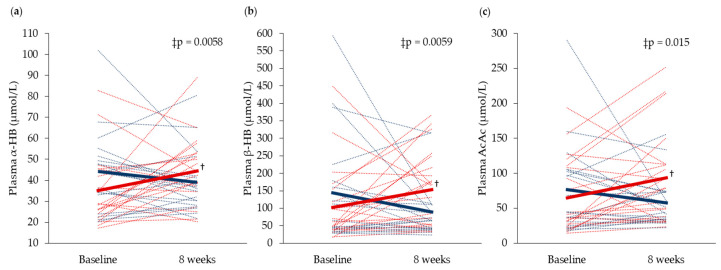
Fasting circulating concentrations of (**a**) α-Hydroxybutyrate (α-HB), (**b**) β-Hydroxybutyrate (β-HB) and (**c**) acetoacetate (AcAc), at baseline and after 8 weeks of supplementation. Concentrations in the Cod-RP group are indicated by blue lines and concentrations in the Control group are indicated by red lines. The means of each group are indicated by bold lines in their respective colors. The results are presented for 19 participants in the Cod-RP group and 21 participants in the Control group. † Within-group changes were tested using the paired samples *T*-test, where *p* < 0.05 was considered significant. ‡ Between-group changes were compared using the independent samples *T*-test.

Fasting serum concentrations of glucose and insulin were unchanged in both groups from baseline to eight weeks (Figure 2). Comparison of serum glucose after OGTT at baseline, and following eight weeks of supplementation, revealed that only glucose concentration at 60 min decreased in the Cod-RP group compared with the Control group (*p* = 0.0078). Changes in postprandial insulin concentrations, from baseline to eight weeks, did not differ between groups at any time points. Fasting plasma concentrations of α-HB, β-HB and AcAc were decreased in the Cod-RP group when compared with the Control group after eight weeks of supplementation (Figure 3).

### 3.4. Plasma and Urine Biomarkers Related to Cod Residual Protein Intake

Plasma and urine concentrations of TMAO were increased, whereas concentrations of creatine, 1-MeHis and 3-MeHis were unchanged in both plasma and urine in the Cod-RP group, when compared with the Control group, after eight weeks intervention (Table 3).

## 4. Discussion

In the current study, we show that eight weeks of cod residual supplementation decreased fasting plasma concentrations of α-HB, β-HB and AcAc, when compared to the Control group. We also observed an increase in TMAO concentration in both plasma and urine in the Cod-RP group, and propose TMAO as a biomarker of cod residual protein intake. To our knowledge, this study is the first to explore the effects of a cod residual protein supplement on glucose regulation and potential biomarkers of cod residual protein intake in lean adults.

The significant reduction in circulating α-HB concentration in the Cod-RP group compared with the Control group suggests that cod residual supplementation may have beneficial effects on glucose regulation, as the concentration of this metabolite is shown to increase under conditions of impaired glucose tolerance [16,17,19]. The current study also demonstrates decreased glucose concentration 60 min after glucose intake in the Cod-RP group, from baseline to eight weeks, compared with the Control group, however, postprandial measurements 30, 90 and 120 min did not differ between the two groups. Previous studies have observed that intake of proteins from cod fillet or cod residuals may improve postprandial glucose regulation in overweight adults and in obese rats, with no effects on fasting serum concentrations of glucose or insulin [12,13,14,38]. In line with this, two clinical studies, investigating the effects of 750 g cod fillet per week in normal-weight adults for four weeks [39], or overweight adults for eight weeks [40], did not find beneficial effects on fasting glucose and insulin concentrations. In the present study cohort, fasting non-esterified fatty acid (NEFA) concentration decreased after Cod-RP supplementation when compared with Control [21], which could be tied to the decrease in α-HB [16,17,19]. α-HB is synthesized in the liver from α-ketobutyrate, which is a substrate for pyruvate dehydrogenase. The pyruvate dehydrogenase activity is indirectly inhibited by increased NEFA concentration [41,42], resulting in more α-ketobutyrate available for α-HB production. The mechanisms underlying the observations in the present study are currently unknown, but based on the pattern of changes, we speculate that the decreased α-HB in the Cod-RP group may be associated with the decrease in circulating NEFA. This is further supported by the decreased concentrations of the ketone bodies β-HB and AcAc in the Cod-RP group, since these are products of NEFA oxidation, and high circulating concentrations of ketone bodies have been associated with impaired glucose tolerance [18]. The decreases in α-HB, β-HB and AcAc suggest that Cod-RP supplementation increased glucose utilization, despite not affecting fasting concentrations of serum glucose and insulin in this healthy cohort.

The increased TMAO concentrations in both plasma and urine in the Cod-RP group is in agreement with studies demonstrating increased urine or plasma/serum TMAO concentrations after cod fillet intake [23,24,25,27]. Indeed, higher urinary TMAO concentrations have been observed in populations with high fish intake, when compared to populations with low fish intake [30,31]. TMAO concentration in plasma and urine have also been shown to increase after intake of foods other than fish, if containing precursors of TMAO [25,26,43], but not to the same extent as after fish intake [25,26,27,44]. Circulating TMAO concentration may increase when kidney function is decreased [45], however, all participants in the current study had normal kidney function, as indicated by normal urine albumin concentration (relative to creatinine) and normal plasma creatinine concentration. Therefore, the increased plasma TMAO concentrations in the Cod-RP group were most likely a result of TMAO intake from the Cod-RP supplements, and not a consequence of impaired kidney function. Following Cod-RP supplementation in the present study, the increased TMAO concentration was more pronounced in urine than in plasma. This is in line with a study demonstrating increased concentrations of TMAO in serum, and particularly in urine, among overweight adults after consumption of 750 g/week of cod fillet for eight weeks [24]. Therefore, this work further suggests that TMAO measured in urine and plasma may be a reliable biomarker of cod intake, and should be measured in future studies assessing compliance to cod residual protein intake.

We found no changes in plasma and urine concentrations of 1-MeHis, 3-MeHis and creatine after Cod-RP supplementation. The concentrations of these biomarkers have been shown to increase after intake of meat [46,47,48,49], which was part of the participants’ habitual dietary intake. This could explain why no differences in the concentrations of 1-MeHis, 3-MeHis and creatine were observed after Cod-RP supplementation, when compared with Control supplementation. However, cod fillet consumption (750 g/week for eight weeks) increased both serum and urine creatine, and 1-MeHis concentrations, compared with meat intake in overweight adults [24]. These contradictory findings may be attributed to a higher intake of creatine and 1-MeHis from consuming 750 g/week of cod fillet, compared with only 57 g/week of cod residual protein in the current study.

The present study has some limitations. The decreases in circulating concentrations of metabolites related to glucose regulation in the current intervention were observed among lean adults, and may not be translatable to individuals at risk of developing type 2 diabetes or cardiovascular disease. Further exploration of changes in concentrations of α-HB, β-HB and AcAc after cod residual protein intake in individuals with impaired glucose tolerance is therefore suggested.

## 5. Conclusions

To conclude, eight weeks of supplementation with cod residual proteins lowered plasma concentrations of the early markers of impaired glucose regulation α-HB, β-HB and AcAc, when compared to the Control group, thus suggesting that cod residual proteins may beneficially affect glucose regulation. TMAO concentrations in plasma, and particularly in urine, were increased after cod residual protein supplementation, indicating that TMAO could be a relevant biomarker of cod residual protein intake.

## Figures and Tables

**Table 1:** Capsule content.

Per Capsule	Cod-RP	Control
Cod residual powder * (mg)	474	0
Microcrystalline cellulose (mg)	42	454
Magnesium stearate (mg)	5.3	4.6
Silica (mg)	5.3	4.6
Total capsule weight (mg)	527	463
Energy ^†^ (kcal)	1.59	0.02

* Crude protein content 64%, total fat content 8.6%. ^†^ Estimated true metabolizable energy. Cod-RP, cod residual protein powder.

**Table 2:** Participant characteristics at baseline.

	Cod-RP (*n* = 19)	Control (*n* = 21)	*p*
	Mean ± SD	Mean ± SD	
Women/Men	7/12	11/10	0.36
Age (years)	28.0 ± 6.9	30.5 ± 7.2	0.28
Body weight (kg)	77.0 ± 16.0	73.1 ± 11.5	0.39
BMI (kg/m^2^)	24.8 ± 2.8	23.8 ± 2.3	0.25
Body fat (%)	19.7 ± 6.8	19.4 ± 6.7	0.89
Body muscle (%)	45.4 ± 4.7	45.4 ± 4.4	0.95
Whole blood HbA1c (mmol/mol)	32.4 ± 2.2	31.5 ± 2.0	0.23
Plasma creatinine (µmol/L)	80.1 ± 13.1	77.2 ± 11.1	0.46
Urine albumin (mg/mmol creatinine)	0.7 ± 0.5	1.0 ± 0.9	0.23
Serum glucose (mmol/L)	5.1 ± 0.4	4.9 ± 0.3	0.21
Serum insulin (pmol/L)	68.1 ± 32.8	56.3 ± 25.9	0.55
Plasma α-HB (µmol/L)	44.2 ± 19.3	35.1 ± 17.0	0.80
Plasma β-HB (µmol/L)	143 ± 156	102 ± 108	0.34
Plasma AcAc (µmol/L)	76.5 ± 67.0	65.1 ± 52.1	0.86
Cigarette/snus * (*n*)	1	2	1.00

Values are presented as mean ± standard deviation. Groups were compared at baseline using independent samples *T*-test for continuous data and Pearson’s Chi-square test for categorical data. * Snus is a Scandinavian tobacco substance which is placed under the upper lip. Cod-RP, cod residual protein powder; BMI, body mass index; HbA1c, glycated haemoglobin; α-HB, α-hydroxybutyrate; β-HB, β-hydroxybutyrate; AcAc, acetoacetate.

**Table 3:** Plasma and urine biomarkers related to cod residual protein intake.

	Baseline	8 Weeks	*p* ^†^	*p* ^‡^
	Mean ± SD	Mean ± SD		
Plasma (µmol/L)				
TMAO				0.048
Cod-RP group	4.3 ± 2.2	5.8 ± 3.3	0.032	
Control group	5.2 ± 6.2	4.6 ± 6.3	0.36	
Creatine				0.11
Cod-RP group	24.9 ± 15.3	26.8 ± 15.4	0.36	
Control group	32.2 ± 18.2	30.5 ± 21.8	0.17	
1-MeHis				0.17
Cod-RP group	13.0 ± 11.5	7.8 ± 7.9	0.16	
Control group	9.2 ± 7.2	10.7 ± 9.1	0.68	
3-MeHis				0.24
Cod-RP group	5.1 ± 1.3	4.9 ± 0.9	0.29	
Control group	4.5 ± 1.0	4.7 ± 1.1	0.29	
Urine (µmol/mmol creatinine)				
TMAO				0.026
Cod-RP group	44.3 ± 22.6	63.2 ± 31.5	0.016	
Control group	53.3 ± 68.3	42.0 ± 43.5	0.30	
Creatine				0.18
Cod-RP group	2.7 ± 4.5	6.6 ± 14.9	0.29	
Control group	7.0 ± 11.8	5.6 ± 11.1	0.37	
1-MeHis				0.49
Cod-RP group	90.9 ± 81.4	54.1 ± 54.0	0.16	
Control group	69.0 ± 62.7	72.1 ± 57.9	0.72	
3-MeHis				0.16
Cod-RP group	29.5 ± 9.4	28.2 ± 6.5	0.77	
Control group	25.1 ± 5.6	26.4 ± 6.7	0.41	

Values are presented as mean ± standard deviation for 19 participants in the Cod-RP group and 21 participants in the Control group. The level of significance was set to < 0.05. ^†^ Within-group changes were tested using paired samples *T*-test. ^‡^ Between-group changes were compared using the independent samples *T*-test. Plasma and urine concentrations of TMAO, creatine, 1-MeHis and 3-MiHis were similar between groups at baseline. Cod-RP, cod residual protein powder; TMAO, Trimethylamine N-oxide; 1-MeHis, 1-Methylhistidine; 3-MeHis, 3-Methylhistidine.
